# Protective Effects of SPA0355, a Thiourea Analogue, Against Lipopolysaccharide-Induced Acute Kidney Injury in Mice

**DOI:** 10.3390/antiox9070585

**Published:** 2020-07-04

**Authors:** Jung-Yeon Kim, Jaechan Leem, Hyo-Lim Hong

**Affiliations:** 1 Department of Immunology, School of Medicine, Catholic University of Daegu, Daegu 42472, Korea; jy1118@cu.ac.kr; 2 Department of Internal Medicine, School of Medicine, Catholic University of Daegu, Daegu 42472, Korea

**Keywords:** sepsis, acute kidney injury, SPA0355, inflammation, oxidative stress, apoptosis

## Abstract

Inflammation and oxidative stress plays an essential role in the pathophysiology of sepsis-associated acute kidney injury (AKI). SPA0355, a thiourea analogue, has been shown to display beneficial effects against a variety of inflammatory diseases arising from its anti-inflammatory and anti-oxidant properties. However, the potential protective effects of SPA0355 against lipopolysaccharide (LPS)-induced AKI have not been explored. The aim of this study was to evaluate the effects of SPA0355 on LPS-induced AKI and investigate its underlying mechanisms. We found that renal dysfunction and histological abnormalities after LPS injection were significantly ameliorated by SPA0355. The compound also reduced renal expression of tubular injury markers. Mechanistically, SPA0355 significantly suppressed plasma and tissue levels of inflammatory cytokines and immune cell infiltration with inhibition of nuclear factor kappa-B p65 signaling. In addition, elevated levels of 4-hydroxynonenal and malondialdehyde after LPS injection were significantly decreased by SPA0355. The compound also regulated expression of pro-oxidant and antioxidant enzymes after LPS injection. Moreover, SPA0355 attenuated LPS-induced tubular cell apoptosis via inhibition of p53 signaling pathway. Altogether, these results suggest that SPA0355 protects against LPS-induced AKI through suppressing inflammation, oxidative stress, and tubular cell apoptosis and might be a potential preventive option for the disease.

## 1. Introduction

Sepsis is a medical condition characterized as a systemic inflammatory response triggered by an infection. If not managed promptly and appropriately, it can lead to multiple organ dysfunction and ultimately death. Acute kidney injury (AKI) is a frequent complication of sepsis in patients hospitalized at an intensive care unit and is closely associated with increased mortality [1,2]. However, current therapy remains reactive and nonspecific, and there is no effective preventive therapy available. Therefore, it is of great clinical importance to develop effective therapeutic approaches to prevent sepsis-associated kidney injury and retard the decline of renal function. Pathogenesis of sepsis-associated AKI is very complex and involves multiple mechanisms. During sepsis, circulating levels of pathogen-associated molecular patterns, such as lipopolysaccharide (LPS), are increased. These inflammatory mediators bind to Toll-like receptors that are expressed on the plasma membrane of immune cells and renal tubular epithelial cells, resulting in increased inflammation, oxidative stress, and tubular cell apoptosis [3,4,5].

**Figure 1:**
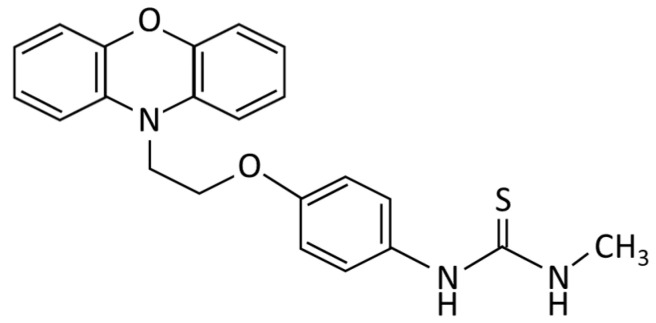
The chemical structure of SPA0355.

SPA0355 (1-methyl-3-[4-(2-phenoxazin-10-ylethoxy)phenyl]thiourea (see Figure 1) is a thiourea analogue that has been shown to exert anti-inflammatory and anti-oxidant actions. The compound effectively ameliorated various inflammatory diseases such as rheumatoid arthritis [6], type 1 diabetes [7], hepatic ischemia/reperfusion injury [8], allergic airway inflammation [9], and postmenopausal osteoporosis [10] in animal models. However, the potential therapeutic effects of SPA0355 on sepsis-associated AKI have not yet been investigated.

An animal model of LPS-induced AKI has been widely used to investigate the mechanisms underlying sepsis-associated AKI and evaluate potential new therapeutic agents for the life-threatening medical condition [11]. In the present study, we aimed to evaluate the potential effects of SPA0355 on LPS-induced AKI and investigate its mechanism of action.

## 2. Materials and Methods

### 2.1. Animal Procedures

C57BL/6N mice (male, 7 weeks of age) were purchased from Samtako Bio Korea (Osan, Korea) and housed at 22 ± 2 °C under a 12 h:12 h light–dark cycle. Standard chow diet and water were provided ad libitum. After 1 week of acclimatization, the mice were randomly assigned to 3 groups: vehicle (Veh; *n* = 8), LPS alone (LPS; *n* = 8) and LPS plus SPA0355 (LPS+SPA; *n* = 8). The LPS group received an intraperitoneal injection of LPS (10 mg/kg body weight; L2880; Sigma-Aldrich, St. Louis, MO, USA). An equal volume of 0.9% saline was intraperitoneally injected into the Veh group. To evaluate the effects of SPA0355 (SML0464; Sigma-Aldrich) on LPS-induced AKI, the LPS+SPA0355 group was intraperitoneally injected with SPA0355 (50 mg/kg body weight in corn oil) 1 h before administration of LPS [12,13]. The dose of SPA0355 was determined based on the results of a previous study [10]. All mice were sacrificed 24 h after LPS injection. Blood samples were collected into anticoagulant-coated tubes by cardiac puncture and kept on ice. Cells were removed from the samples by centrifugation for 10 min at 1000× *g* using a refrigerated centrifuge. The resulting plasma supernatants were used for subsequent biochemical analysis, which was performed approximately 2 h after collection. Kidneys were rapidly isolated and immediately fixed in 4% paraformaldehyde. Efforts were made to minimize temporal differences in the collection and testing of blood and tissue samples between groups of mice. All experimental procedures were approved by the Institutional Animal Care and Use Committee of the Daegu Catholic University Medical Center (DCIAFCR-191112-08-Y).

### 2.2. Biochemical Analysis

Plasma levels of creatinine were analyzed using a creatinine assay kit (DICT-500; Bioassay Systems, Hayward, CA, USA). Plasma levels of blood urea nitrogen (BUN) were measured using a BUN assay kit (AM165; Asan Pharmaceutical, Seoul, Korea). Plasma levels of tumor necrosis factor-α (TNF-α) and interleukin-6 (IL-6) were analyzed using ELISA kits (MTA00B and M6000B; R&D Systems, Minneapolis, MN, USA). Renal levels of malondialdehyde (MDA) were measured using a lipid peroxidation assay kit (MAK085; Sigma-Aldrich). All assays were performed according to the manufacturer’s instructions.

### 2.3. Histological Analysis

Kidney tissues were dehydrated in graded ethanol solutions, cleared in xylene, and embedded in paraffin. The sections were stained with hematoxylin & eosin (H&E) stain and periodic acid Schiff (PAS) stain. Tubular injury in PAS-stained sections was analyzed in 10 randomly chosen fields per each kidney at ×400 magnification. The tubular injury score was calculated based on the percentage of damaged area, as follows: 0, 0%; 1, ≤10%; 2, 11–25%; 3, 26–45%; 4, 46–75%; and 5, 76–100% [14,15]. In addition, outer diameters of proximal and distal tubules were measured in 30 randomly chosen tubules per each kidney at ×400 magnification. ImageJ software (NIH, Bethesda, MD, USA) was used for quantitative analysis of the renal tubular diameter.

To identify the brush border of proximal tubules, the sections were stained with fluorescein-labeled lotus tetragonolobus lectin (LTL; FL-1321-2; Vector Laboratories, Burlingame, CA, USA). For immunohistochemistry (IHC), the sections were incubated with primary antibodies against neutrophil gelatinase-associated lipocalin (sc-515876; NGAL; Santa Cruz Biotechnology, Santa Cruz, CA, USA), kidney injury molecule-1 (Kim-1; ab47635; Abcam, Cambridge, MA, USA), Mac-2 (ab2785; Abcam), CD4 (ab183685; Abcam), or 4-hydroxynonenal (4-HNE; ab46545; Abcam) overnight and then probed with a secondary antibody. Hematoxylin was used as the counter-stain. The percentage of positively stained area was assessed in 10 arbitrarily chosen fields per each kidney at ×400 magnification using i-Solution DT software (IMTechnology, Vancouver, BC, Canada). Mac-2 or CD4-stained cells were counted in 10 arbitrarily chosen fields per each kidney at ×400 magnification.

### 2.4. Western Blot Analysis

Western blotting was performed as previously described [16]. Briefly, protein samples extracted from kidneys were loaded onto sodium dodecyl sulfate polyacrylamide gradient gels (Thermo Fisher Scientific, Waltham, MA, USA) and then transferred onto nitrocellulose membranes (GE Healthcare, Chicago, IL, USA). The membranes were probed with a specific primary antibody overnight. After washing, the membrane was incubated with goat anti-rabbit IgG (#7074; Cell Signaling, Danvers, MA, USA) or horse anti-mouse IgG antibody (#7076; Cell Signaling) conjugated to horseradish peroxidase. The primary antibodies used in this study were as follows: anti-Bax (sc-7480; Santa Cruz Biotechnology), anti-cleaved caspase-3 (#9661; Cell Signaling), anti-IL-6 (ab208113; Abcam), anti-inhibitor κB-α (IκB-α; #9242; Cell Signaling), anti-p-IκB-α (#2859; Cell Signaling), anti-NGAL (sc-515876; Santa Cruz Biotechnology), anti-nicotinamide adenine dinucleotide phosphate oxidase 4 (NOX4; NB110-58849; Novus Biologicals, Littleton, CO, USA), anti-nuclear factor-κB (NF-κB) p65 (#8242; Cell Signaling), anti-p-NF-κB p65 (#3033; Cell Signaling), anti-p53 (#2524; Cell Signaling), anti-cleaved poly(ADP-ribose) polymerase-1 (cleaved PARP-1; #94885; Cell Signaling), anti-TNF-α (ab1793; Abcam), and anti-glyceraldehyde-3-phosphate dehydrogenase (GAPDH; #2118; Cell Signaling) antibody. GAPDH was used as a loading control. The signals were detected using an image analyzer (ChemiDoc^TM^ XRS+ System; Bio-Rad Laboratories).

### 2.5. Real-Time Reverse Transcription-Polymerase Chain Reaction (RT-PCR)

Total RNA was isolated from kidneys using the RNeasy Mini Kit (74104; Qiagen, Valencia, CA, USA). The RNA was reversed transcribed into cDNA using the High-Capacity cDNA Reverse Transcription Kit (4368814; Applied Biosystems, Foster City, CA, USA). Target cDNA levels were quantified by real-time RT-PCR using the Real-Time PCR 7500 system (Applied Biosystems) and the Power SYBR Green PCR Master Mix (4368577; Applied Biosystems). Primer sets used in this study are listed in Table 1. The relative expression levels of each gene were normalized to the GAPDH gene.

### 2.6. TdT-Mediated dUTP Nick End Labeling (TUNEL) Staining

Apoptosis was analyzed using the in situ cell death detection kit (11684795910; Roche Diagnostics, Indianapolis, IN, USA). Briefly, kidney sections were deparaffinized in xylene, rehydrated in graded ethanol solutions, and permeabilized. After washing, a TUNEL reaction mixture was added to the sections, which were then incubated for 1 h at 37 °C. Nuclei were visualized using 4′, 6-diamidino-2-phenylindole (DAPI) staining. TUNEL-positive cells were counted in 10 randomly chosen fields per each kidney at ×400 magnification.

### 2.7. Statistical Analysis

Data are expressed as the mean ± standard error of the mean (SEM). Statistical analysis was performed using one-way analysis of variance with *post hoc* Bonferroni’s tests. Statistical significance was defined as *p* < 0.05.

## 3. Results

### 3.1. SPA0355 Ameliorated LPS-Induced Kidney Damage

**Figure 2:**
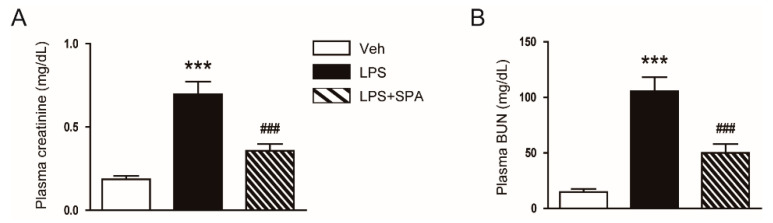
SPA0355 ameliorated renal dysfunction in mice treated with lipopolysaccharide (LPS). Mice were treated with a single intraperitoneal injection of SPA0355 (50 mg/kg body weight in corn oil) 1 h before administration of LPS (10 mg/kg body weight). (**A**) Plasma levels of creatinine. (**B**) Plasma levels of blood urea nitrogen (BUN). Results are from 8 mice per group (biological replicates) and 2 technical replicates per mouse. *** *p* < 0.001 vs. vehicle-treated mice (Veh). ^###^ *p* < 0.001 vs. LPS-injected mice (LPS).

Administration of SPA0355 significantly improved renal dysfunction in LPS-treated mice, as reflected by reduced plasma levels of creatinine and BUN (Figure 2A,B).

**Figure 3:**
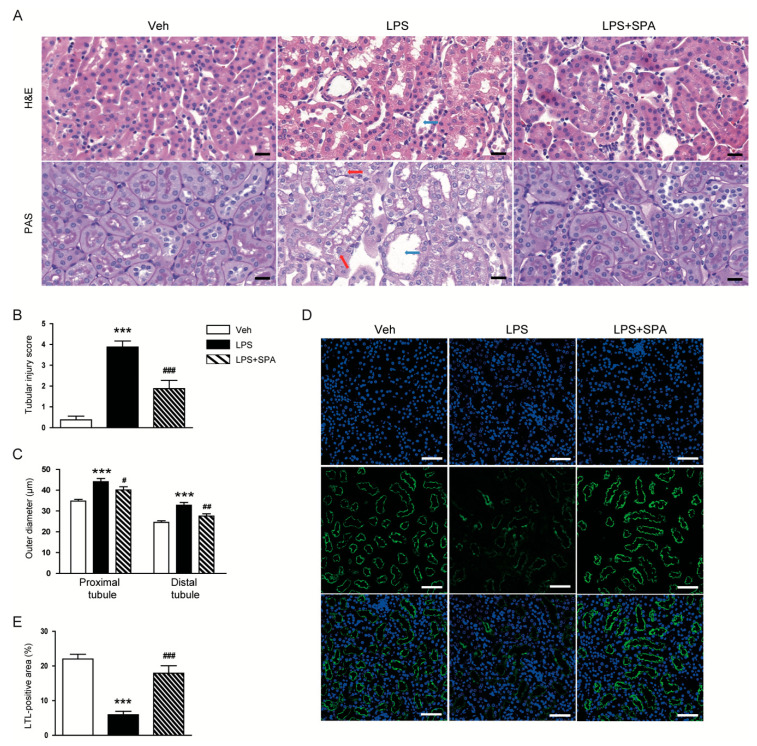
SPA0355 attenuated histological abnormalities in LPS-treated mice. (**A**) Representative images of Hematoxylin and eosin (H&E) and periodic acid Schiff (PAS) staining in kidneys. Red arrows indicate vacuolar degeneration. Blue arrows indicate tubular dilatation. Bar = 20 μm. (**B**) Tubular injury score. (**C**) Outer diameters of proximal and distal tubules. (**D**) Representative images of lotus tetragonolobus lectin (LTL) staining in kidneys. Bar = 50 μm. (**E**) Percentage of LTL-positive area per field. Results are from 8 mice per group (biological replicates) and 2 technical replicates per mouse. *** *p* < 0.001 vs. vehicle-treated mice (Veh). ^#^ *p* < 0.05, ^##^ *p* < 0.01, and ^###^ *p* < 0.001 vs. LPS-injected mice (LPS).

Moreover, renal histological abnormalities, as represented by increased tubular injury score, were observed in the kidneys of LPS-treated mice (Figure 3A,B). Outer diameters of proximal and distal tubules were longer in the LPS-treated mice than in the vehicle-treated control mice (Figure 3C). LTL staining also revealed that LPS-treated mice displayed loss of brush border in proximal tubules (Figure 3D,E). However, all these histological abnormalities induced by LPS were significantly attenuated by SPA0355.

**Figure 4:**
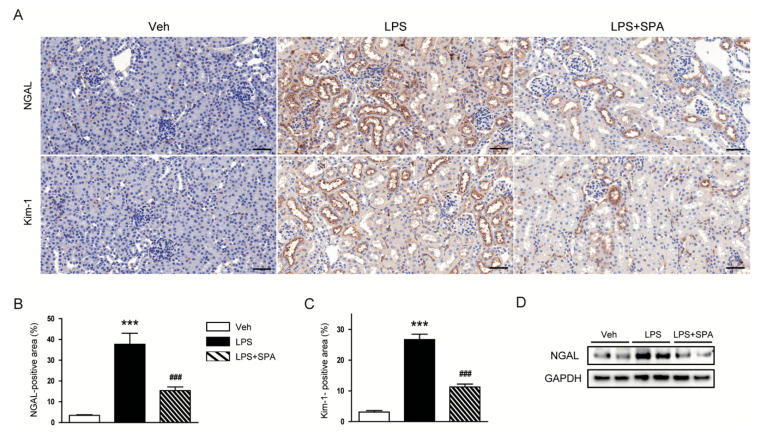
SPA0355 attenuated LPS-induced tubular injury. (**A**) Representative images of immunohistochemistry (IHC) of neutrophil gelatinase-associated lipocalin (NGAL) or kidney injury molecule-1 (Kim-1) in kidneys. Bar = 50 μm. (**B**) Percentage of NGAL-positive area. (**C**) Percentage of Kim-1-positive area. (**D**) Representative images of Western blotting of NGAL and glyceraldehyde-3-phosphate dehydrogenase (GAPDH). Results are from 8 mice per group (biological replicates) and 2 technical replicates per mouse. *** *p* < 0.001 vs. vehicle-treated mice (Veh). ^###^ *p* < 0.001 vs. LPS-injected mice (LPS).

To further evaluate the effects of SPA0355 on LPS-induced tubular injury, the kidneys sections were stained with an antibody against NGAL or Kim-1. IHC staining revealed that SPA0355 significantly attenuated elevated expression of both markers of tubular injury after LPS injection (Figure 4A–C). Inhibitory effect of the compound on renal expression of NGAL was confirmed by Western blot analysis (Figure 4D).

### 3.2. SPA0355 Suppressed LPS-Induced Inflammatory Responses

**Figure 5:**
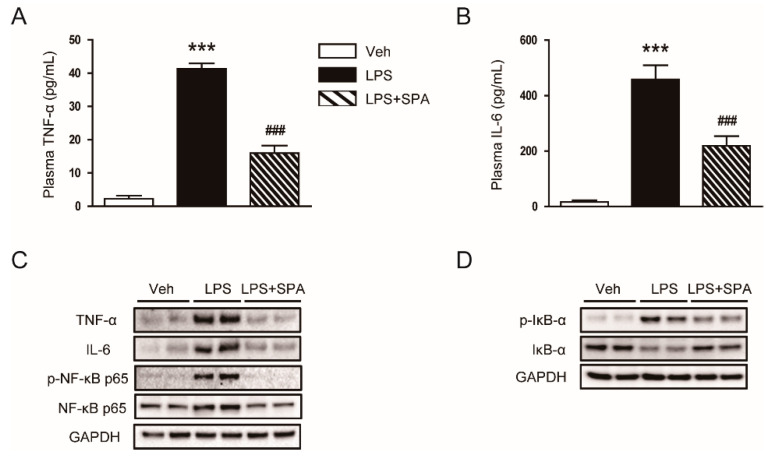
SPA0355 decreased plasma and tissue levels of inflammatory cytokines in LPS-treated mice. (**A**) Plasma tumor necrosis factor-α (TNF-α). (**B**) Plasma interleukin-6 (IL-6). (**C**) Representative images of Western blotting of TNF-α, IL-6, p-nuclear factor-κB (NF-κB) p65, NF-κB p65, and GAPDH. (**D**) Representative images of Western blotting of p-IκB-α, IκB-α, and GAPDH. Results are from 8 mice per group (biological replicates) and 2 or 3 technical replicates per mouse. *** *p* < 0.001 vs. vehicle-treated mice (Veh). ^###^ *p* < 0.001 vs. LPS-injected mice (LPS).

It is well known that LPS can induce acute inflammatory responses by inducing the release of various inflammatory cytokines from immune cells, including macrophages and CD4^+^ T cells [3,4,5]. In addition, SPA0355 has been shown to exert anti-inflammatory action [6,7,8,9,10]. Therefore, we examined the effects of SPA0355 on LPS-induced inflammation. As expected, LPS-treated mice displayed elevated levels of TNF-α and IL-6 in both plasma (Figure 5A,B) and kidney tissues (Figure 5C). Moreover, levels of p-NF-κB p65 and NF-κB p65 were increased after LPS injection (Figure 5C). LPS treatment also increased phosphorylation and degradation of IκB-α (Figure 5D). However, all these inflammatory responses induced by LPS were largely attenuated by SPA0355.

**Figure 6:**
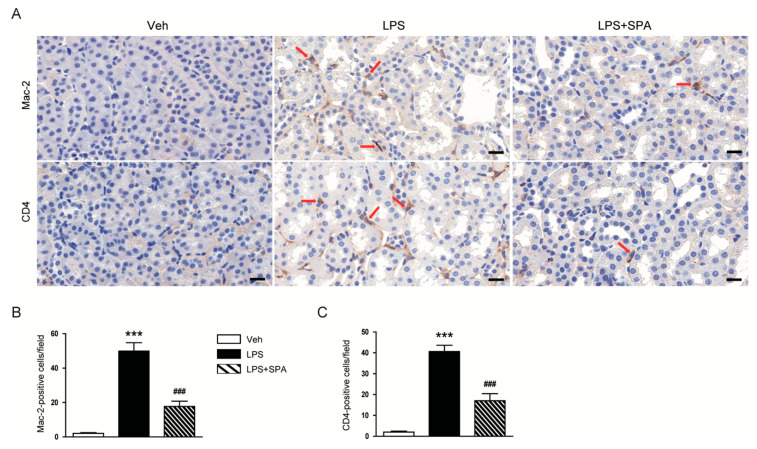
SPA0355 prevented immune cell infiltration into damaged kidneys and inhibited NF-κB activation. (**A**) Representative images of IHC of Mac-2 or CD4 in kidneys. Red arrows indicate positively stained cells. Bar = 20 μm. (**B**) Number of Mac-2-positive cells. (**C**) Number of CD4-positive cells. Results are from 8 mice per group (biological replicates) and 2 technical replicates per mouse. *** *p* < 0.001 vs. vehicle-treated mice (Veh). ^#^^##^ *p* <0.001 vs. LPS-treated mice (LPS).

IHC staining also revealed that administration of SPA0355 significantly suppressed infiltration of macrophages and CD4^+^ T cells, as evidenced by decreased number of Mac-2 or CD4-positive cells respectively, into damaged kidneys of mice treated with LPS (Figure 6A–C).

### 3.3. SPA0355 Attenuated LPS-Induced Oxidative Stress

**Figure 7:**
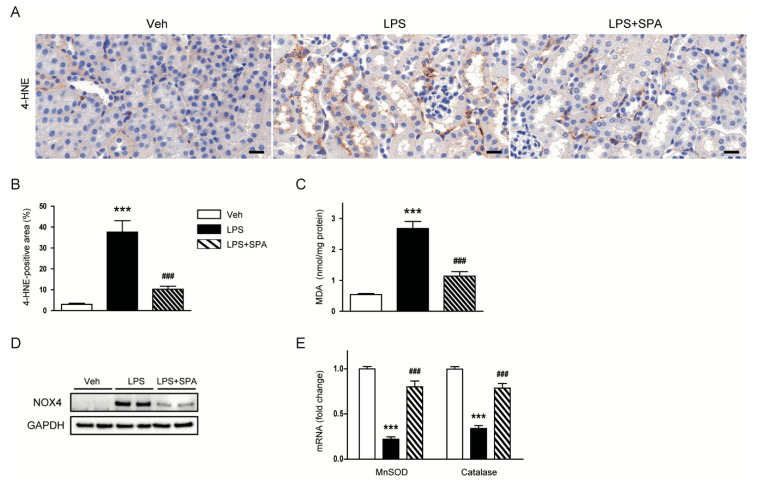
SPA0355 decreased renal oxidative stress and regulated levels of pro-oxidant and antioxidant enzymes in LPS-treated mice. (**A**) Representative images of IHC of 4-hydroxynonenal (4-HNE) in kidneys. Bar = 20 μm. (**B**) Percentage of 4-HNE-positive area per field. (**C**) Renal levels of malondialdehyde (MDA). (**D**) Representative images of Western blotting of nicotinamide adenine dinucleotide phosphate oxidase 4 (NOX4) and GAPDH. (**E**) The mRNA levels of manganese superoxide dismutase (MnSOD) and catalase. Results are from 8 mice per group (biological replicates) and 2 or 3 technical replicates per mouse. *** *p* < 0.001 vs. vehicle-treated mice (Veh). ^###^ *p* <0.001 vs. LPS-injected mice (LPS).

Oxidative stress plays a critical role in the development of LPS-induced AKI [3,4,5]. SPA0355 has also been shown to have anti-oxidant properties [8]. Therefore, we next evaluated the effects of SPA0355 on renal oxidative stress induced by LPS. IHC staining with anti-4-HNE antibody revealed that 4-HNE-stained area was largely increased in LPS-treated mice compared to vehicle-treated control mice (Figure 7A,B). LPS-treated mice also displayed elevated renal levels of MDA (Figure 7C). However, all these changes were significantly attenuated by administration of SPA0355. In addition, we found that elevated protein levels of NOX4 after LPS injection were markedly reduced by SPA0355 (Figure 7D). Reduced mRNA expression of MnSOD and catalase after LPS injection was also significantly restored by SPA0355 (Figure 7E).

### 3.4. SPA0355 Inhibited LPS-Induced Tubular Cell Apoptosis

**Figure 8:**
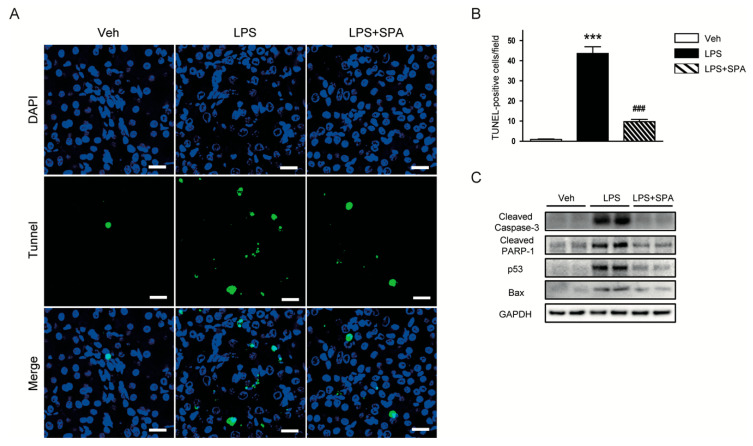
SPA0355 reduced tubular cell apoptosis in LPS-treated mice. (**A**) Representative images of terminal deoxynucleotidyl transferase-mediated deoxyuridine triphosphate nick end labeling (TUNEL) staining in kidneys. Bar = 20 μm. (**B**) Number of TUNEL-positive cells. (**C**) Representative images of Western blotting of cleaved caspase-3, cleaved poly(ADP-ribose) polymerase-1 (PARP-1), p53, Bax, and GAPDH. Results are from 8 mice per group (biological replicates) and 2 or 3 technical replicates per mouse. *** *p* < 0.001 vs. vehicle-treated mice (Veh). ^###^ *p* < 0.001 vs. LPS-injected mice (LPS).

Tubular cell apoptosis is also an important pathogenic process in LPS-induced AKI [3,4,5]. Thus, we next evaluated the effects of SPA0355 on LPS-induced apoptotic cell death. LPS-treated mice exhibited increased number of TUNEL-stained cells in the kidneys (Figure 8A,B). However, LPS-induced tubular cell apoptosis was significantly attenuated by administration of SPA0355. In addition, Western blot analysis showed that SPA0355 significantly reduced protein levels of cleaved caspase-3, cleaved PARP1, p53, and Bax (Figure 8C).

## 4. Discussion

**Figure 9:**
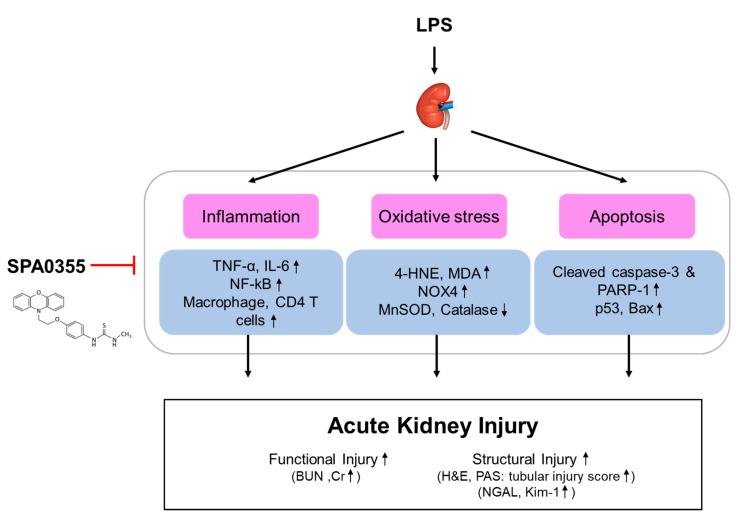
A graphical representation of the mechanism for the protective effects of SPA0355 on LPS-induced acute kidney injury. SPA0355 ameliorated LPS-induced functional and structural renal injury. These beneficial effects of SPA0355 were attributed to the suppression of inflammation, oxidative stress, and apoptosis.

Previous studies have shown that some urea and thiourea analogues have anti-inflammatory effects [17,18,19]. Among them, SPA0355, a synthetic thiourea analogue, was identified as a potent inhibitor of NF-κB activity [19]. Subsequent animal studies have also demonstrated that the compound exerts therapeutic effects against various inflammatory diseases [6,7,8,9,10]. However, the potential effects of SPA0355 on LPS-induced AKI have not yet been investigated. In this study, we found that administration of SPA0355 alleviated renal dysfunction and histological abnormalities in LPS-treated mice. These effects of the compound were associated with inhibition of inflammatory responses, oxidative stress, and tubular cell apoptosis (Figure 9).

Sepsis-associated AKI is a common complication in patients at an intensive care unit and is associated with high mortality [1,2]. Although many efforts have addressed development of specific interventions for preventing the disease, there is no effective preventive therapy currently available. In the present study, we found that administration of SPA0355 attenuated renal dysfunction, as reflected by decreased plasma concentrations of creatinine and BUN, and histological abnormalities such as tubular dilatation, vacuolar degeneration, and brush border loss. In addition, elevated expression of tubular injury markers NGAL and Kim-1 was also largely reduced by SPA0355. Taken together, these results indicate that SPA0355 protects from LPS-induced renal functional and structural injury.

The mechanism by which sepsis causes AKI is very complex and contains multiple processes. Among them, inflammation is considered a key process for the development of sepsis-associated AKI [3,4,5]. LPS, also known as endotoxin, is the main component of the outer membrane of Gram-negative bacteria and its circulating level is increased during sepsis. This molecule binds to Toll-like receptors that are presented on the surface of immune cells and renal tubular epithelial cells, resulting in the excessive release of inflammatory mediators [3,4,5]. In the present study, we measured plasma and renal levels of TNF-α and IL-6 to evaluate the effects of SPA0355 on LPS-induced inflammation. LPS-treated mice exhibited increased plasma and renal concentrations of these inflammatory cytokines compared to vehicle-treated control mice, indicating that the endotoxin induces systemic and local inflammatory responses. However, administration of SPA0355 significantly reduced the increased levels of the cytokines in both plasma and kidney tissue. Previous studies also showed that massive infiltration of immune cells, such as macrophages and CD4^+^ T cells, into the kidneys is a common feature of LPS-induced AKI [20,21]. We observed that administration of SPA0355 reduced accumulation of macrophages and CD4^+^ T cells in the kidneys of LPS-treated mice. It is well known that production of inflammatory mediators in the injured kidneys is mainly regulated by NF-κB [22,23]. SPA0355 was originally identified as a potent NF-κB inhibitor and has been shown to effectively suppress NF-κB activity in various animal models for inflammatory diseases [6,7,8,9,10]. Therefore, to gain a further insight into the mechanism by SPA0355 prevents LPS-induced inflammatory responses, we next examined its effect on NF-κB signaling pathway. We found that increased protein levels of p-NF-κB p65 and NF-κB p65 after LPS injection were largely attenuated by SPA0355. Moreover, SPA0355 suppressed the increased phosphorylation and degradation of IκB-α induced by LPS. It is well known that in the basal state, NF-kB binds to IκB-α and is sequestered in the cytosol. Upon stimulation, IκB-α is phosphorylated by IkB kinases. Phosphorylated IkB is subsequently ubiquitinated and degraded by the proteasomal pathway, thus allowing NF-kB to translocate into the nucleus, where it modulates the expression of inflammatory target genes [24]. Altogether, these results suggest that SPA0355 exerts anti-inflammatory effects during sepsis.

Accumulating evidence suggests that oxidative stress also plays a crucial role in sepsis-associated AKI [3,4,5]. LPS induces excessive production of reactive oxygen species (ROS) and thereby lipid peroxidation, resulting in aggravation of tissue injury [25]. In the present study, we stained the kidney sections with an antibody against 4-HNE, a product of lipid peroxidation, and measured renal levels of MDA, another marker of lipid peroxidation. We observed that LPS-treated mice exhibited elevated renal levels of 4-HNE and MDA. However, these effects of LPS were effectively attenuated by SPA0355. Our findings were supported by a previous study showing that the compound suppresses oxidative stress induced by hepatic ischemia/reperfusion in mice [8]. We also found that SPA0355 markedly reversed elevated NOX4 expression in the kidneys of LPS-treated mice. NOX4 is one of the major sources of ROS in the pathogenesis of LPS-induced AKI [26,27]. Thus, downregulation of the pro-oxidant enzyme by SPA0355 is presumably involved in the suppression of ROS generation and subsequent oxidative tissue damage. We also found that SPA0355 significantly restored reduced renal expression of MnSOD and catalase in LPS-treated mice. Taken together, these results suggest that SPA0355 suppressed LPS-induced oxidative stress through suppressing ROS generation and activating antioxidant systems.

Previous studies have shown that antioxidants, such as N-acetylcysteine, mitoQ, and plastoquinol decylrhodamine 19, effectively ameliorated LPS-induced organ dysfunction mainly through inhibiting oxidative stress and inflammation [28,29,30]. Because ROS can activate NF-kB [31], it was considered that suppressing ROS accumulation resulted in inhibition of NF-kB activity and resultant inflammatory responses. On the other hand, SPA0355 was originally identified as a potent NF-κB inhibitor [19]. Accumulating evidence suggest that some NF-kB-regulated genes play a critical role in regulating the intracellular amount of ROS [32]. Thus, inhibition of NF-κB activity by SPA0355 might, at least in part, contribute to its suppressive effect on oxidative stress.

Besides inflammation and oxidative stress, apoptotic cell death of tubular epithelial cells is also involved in the pathogenesis of sepsis-associated AKI [3,4,5]. LPS can induce apoptosis in renal tubular epithelial cells [33,34]. A previous study showed that treatment with a pan-caspase inhibitor significantly reduced apoptotic cell death and renal damage in LPS-induced AKI [35]. In the present study, we found that LPS-treated mice exhibited elevated number of TUNEL-positive cells with caspase-3 activation in the kidneys compared to vehicle-treated control mice. Protein levels of p53 and its transcriptional target, pro-apoptotic Bax, were also increased. However, all these effects of LPS were largely attenuated by SPA0355. In line with our findings, treatment with SPA0355 before hepatic ischemia/reperfusion injury significantly reduced apoptotic cell death and concordantly changed the expression of apoptotic proteins in mice [8]. Collectively, these results suggest that SPA0355 attenuated LPS-induced tubular cell apoptosis through suppressing p53 signaling pathway.

## 5. Conclusions

In conclusion, our findings suggest that SPA0355 prevents LPS-induced AKI through suppression of inflammation, oxidative stress, and tubular cell apoptosis. The compound might be a potential protective agent against sepsis-associated AKI. To improve the clinical significance of our findings, further studies will be needed to evaluate whether administration of SPA0355 after LPS injection also has a therapeutic effect against the disease.

## Figures and Tables

**Table 1:** Primer sets for real-time RT-PCR.

Gene	Primer Sequence (5′→3′)	Accession No.
MnSOD ^1^	Forward: AACTCAGGTCGCTCTTCAGC Reverse: CTCCAGCAACTCTCCTTTGG	NM_013671.3
Catalase	Forward: CAAGTACAACGCTGAGAAGCCTAAG Reverse: CCCTTCGCAGCCATGTG	NM_009804.2
GAPDH ^2^	Forward: ACTCCACTCACGGCAAATTC Reverse: TCTCCATGGTGGTGAAGACA	NM_001289726.1

**^1^** Manganese superoxide dismutase. **^2^** Glyceraldehyde-3-phosphate dehydrogenase.
